# Intractable Vomiting in the Setting of Glucagon-Like Peptide-1 (GLP-1) Receptor Agonist and Cannabis Usage

**DOI:** 10.7759/cureus.97851

**Published:** 2025-11-26

**Authors:** Peter DesRochers, Linus Kaiser, Seoyoon Lee, Garrett A Perchetti, Roxana Lazarescu

**Affiliations:** 1 Internal Medicine, Medical University of the Americas, Potworks , KNA; 2 Internal Medicine, Touro College of Osteopathic Medicine, New York, USA; 3 Internal Medicine, Wyckoff Heights Medical Center, Brooklyn, USA

**Keywords:** adult gastroenterology, adverse drug reaction, drug intolerance, dual incretin therapy, gastrointestinal tract adverse effect, gip and glp-1 receptor agonist, glp-1 receptor agonists, obesity, tirzepatide

## Abstract

The prevalence of type 2 diabetes mellitus has increased drastically over the past century. In the past few years, Glucagon-Like Peptide-1 (GLP-1) receptor agonists have gained significant popularity for medical management of type 2 diabetes mellitus, especially in patients with comorbid conditions. In this report, we describe the case of a 55‑year‑old woman with a history of chronic kidney disease, diabetes mellitus type 2 with neuropathy, peripheral vascular disease, and prior pancreatitis, who presented with severe gastrointestinal symptoms, sepsis physiology, and evidence of metabolic derangement shortly after up‑titration of tirzepatide therapy, consistent with the established temporal relationship of GLP-1 dose titration and gastrointestinal side effects. Her course was further complicated by gastrointestinal bleeding, hemodynamic instability, and the need for multidisciplinary management.

This case highlights important diagnostic considerations related to the gastrointestinal adverse effects of incretin-based therapies and underlines the challenges of managing complex, multimorbid patients with overlapping clinical syndromes. This case outlines the importance of a multidisciplinary approach, including endocrinology, gastroenterology, and cardiology, in order to manage complex adverse drug reactions.

## Introduction

The prevalence of type 2 diabetes mellitus has continuously increased throughout the 21st century, affecting nearly 16% of the United States population as of 2023 [1]. Given its vast prevalence throughout the country, type 2 diabetes and its complications carry a large economic burden and place a significant strain on the US healthcare system [2]. In recent years, Glucagon-Like Peptide-1 (GLP-1) receptor agonists have gained popularity as a key medication in patients' diabetes management regimens, particularly in those patients with comorbid conditions like chronic kidney disease and cardiovascular disease [3]. Drugs like tirzepatide, a dual incretin agonist that binds both the glucose-dependent insulinotropic polypeptide and glucagon-like peptide receptors, have been shown to lead to enhanced weight loss effects and glycemic control when compared to other GLP-1 medications [4,5]. 

However, most GLP-1 agonizing drugs have been associated with dose-dependent side effects, most commonly including gastrointestinal upset, including nausea, vomiting, and diarrhea [4]. In this report, we describe the case of a 55‑year‑old woman with a history of chronic kidney disease (CKD), diabetes mellitus type 2 with neuropathy, peripheral vascular disease, and prior pancreatitis, who presented with severe gastrointestinal symptoms, sepsis physiology, and evidence of metabolic derangement shortly after up‑titration of tirzepatide therapy. A key differential diagnosis in this case is cannabis hyperemesis syndrome, which has a similar vague presentation of nausea, vomiting, and abdominal pain [6]. This case demonstrates the need for further research to more accurately distinguish the source of these gastrointestinal symptoms.

## Case presentation

A 55-year-old female with a past medical history of CKD, acute kidney injury (AKI) superimposed on CKD, diabetes mellitus type 2 with diabetic neuropathy, pancreatitis, chronic obstructive pulmonary disease, tubal ligation, rectocele, and peripheral vascular disease (PVD) presented to the Emergency Department (ED) with vomiting, diarrhea, and weakness for the prior three days. She also complained of mild abdominal pain (8/10 in severity) that was non-radiating in the emergency department, and had one episode of emesis during triage. She stated that she had multiple episodes of vomiting every day over the previous three days and was unable to tolerate any oral food or drinks, including water, as a result. The patient denied seeing any red blood in her vomit but stated that sometimes it is a dark brown color. She endorsed recent dry heaving because there was no food left in her system. She stated that the abdominal pain was located in the epigastric region and denied any trigger for the pain. The patient was an active tobacco user, smoking four packs per day, and endorsed smoking cannabis daily to help her relax.

During the first day of admission, the patient stated that she had a recent change to her medication; she was on tirzepatide once per week before stopping for two months. She restarted her medication recently on June 4th, 2025, at 2.5 mg, and was increased to 5 mg four weeks later, and was then increased again to 7.5 mg in August; her symptoms started two days after this last adjustment. Tirzepatide and empagliflozin were held due to these symptoms and were replaced with a weight-based insulin regimen for glycemic control. The patient denied any fever, chest pain, headache, shortness of breath, palpitations, melena, hematochezia, or any numbness in her extremities. She also denied any recent travel or antibiotic use. On the physical exam, epigastric tenderness was noted as well as a negative guaiac test. Vital signs included a temperature of 99℉, tachycardic pulse of 128 beats per minute (bpm), respiratory rate of 14 breaths per minute, oxygen saturation of 96%, and an elevated blood pressure of 184/89 mmHg. Gastroenteritis was suspected, as well as further evaluation for associated metabolic derangements vs dehydration. Sepsis workup was initiated, and labs were relevant for elevated troponin (59.1) and lactate (3.6). CTAP showed wall thickening of multiple small bowel loops and colon, suggestive of enterocolitis. Urine toxicology was positive for marijuana. In the ED, she received 3 L of intravenous (IV) fluids, metoclopramide 10 mg, and ondansetron 4 mg. 

**Figure 1:**
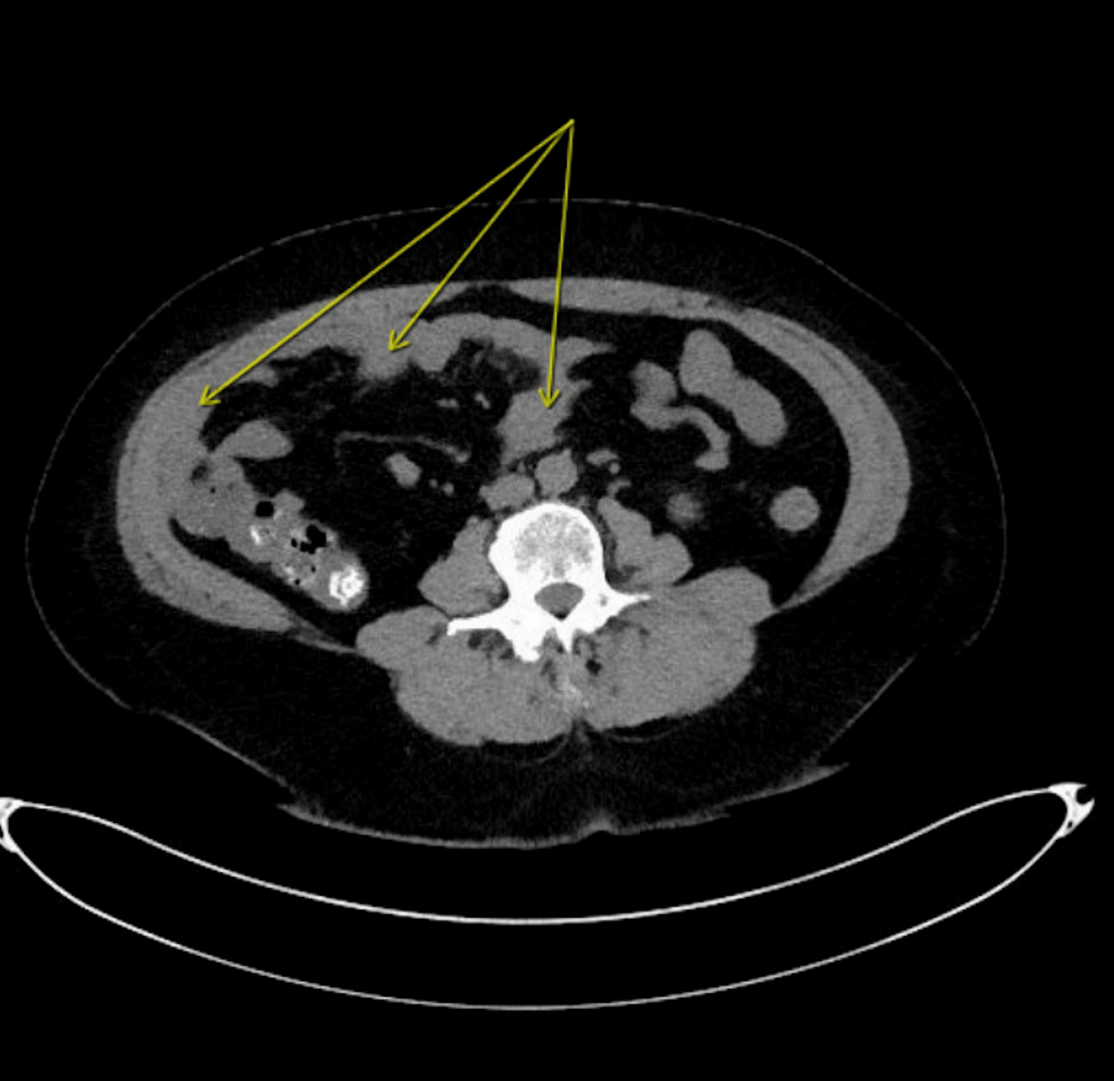
CT of the abdomen and pelvis without contrast Imaging demonstrates wall thickening of multiple small bowel loops (arrows) suggestive of enterocolitis.

She was planned to be admitted to telemetry for continuation of IV hydration, antibiotics, and serial abdominal exams. She was admitted for severe sepsis secondary to complicated urinary tract infection (UTI) vs enterocolitis. On the first day of admission, she was given ciprofloxacin 400 mg IV twice a day (BID) and metronidazole 500 mg IV thrice a day (TID) for urinary tract infection (UTI) and enterocolitis. Due to the positive troponin, a statin loading dose of aspirin (325 mg) was given with subsequent aspirin 81 mg daily. Cardiology was consulted, and an echocardiogram was performed to rule out cardiac pathology. Because the patient had AKI superimposed on CKD, fluid hydration was given. CT of the abdomen and pelvis showed wall thickening of multiple small bowel loops and colon, suggestive of enterocolitis (Figure 1).

The next day, urine toxicology was positive for tetrahydrocannabinol (THC), consistent with cannabis use. She continued to have poor oral intake and was mostly on a fluid diet. She was also started on ondansetron every four hours as needed due to persistent nausea. Doppler was performed due to chronic peripheral vascular disease (PVD) and showed >50% stenosis of the distal peroneal artery. The morning of hospital day three, she reported one episode of vomiting with low appetite. In the afternoon, she was able to tolerate clear liquids and did not have any more episodes of vomiting. 

On day four of admission, during rounds, the patient admitted to feeling worse overnight. She endorsed some odynophagia, dyspnea, a cough with ambulation, and reported two episodes of soft, black, large bowel movements overnight and in the morning. She denied any chest pain or palpitations, although tachycardia was noted on telemetry monitoring. Heparin, aspirin, and clopidogrel were held due to the bleeding, and ondansetron was changed to cefazolin and metronidazole to complete a seven-day course of antibiotics. Transthoracic echocardiogram results came back showing a normal left ventricular ejection fraction, but did indicate diastolic dysfunction. Cardiology recommended no acute intervention at that time. Due to the dyspnea and tachycardia, D-dimers were ordered and came back positive at 947 ng/mL. Venous duplex doppler of the bilateral lower extremities was ordered with a ventilation-perfusion (VQ) scan. 

Anticoagulation was held at that point and reassessed after the venous doppler came back negative and the VQ scan resulted in a low probability of a pulmonary embolism. During the 5th day of hospitalization, she reported some melena during the previous overnight; the fecal occult bleeding test was positive. She also said she had some dyspnea and generalized weakness, but did not have any vomiting or throat pain. Lactic acidosis had also improved from 3.6 to 1.7. 

On her 6th and last day of hospitalization, she reported no nausea, vomiting, epigastric discomfort, or melena for the past 12 hours. Her shortness of breath resolved, and she was without coughing. Labs such as complete blood count (CBC), comprehensive metabolic panel (CMP), lipase, procalcitonin, and lactic acid were trending in the right direction, and she was able to tolerate food without any pain or difficulty swallowing (Tables 1, 2). Cardiology and gastroenterology cleared the patient for discharge, and she was discharged on insulin glargine, amlodipine, sucralfate, protonix, and ReLion NovoLog® flexpen, and followed up in a week with the medicine clinic. She was also scheduled to follow up with outpatient gastroenterology, nephrology, and cardiology appointments

**Table 1:** Comprehensive metabolic panel from day 6 of admission SGOT: Serum glutamic-oxaloacetic transaminase, AST: Aspartate aminotransferase, SGPT: Serum glutamic pyruvic transaminase, ALT: Alanine aminotransferase, eGFR: Estimated glomerular filtration rate.

Test Parameter	Result	Direction of Change	Common Adult Reference Range	Units
Calcium Level	9.5	Normal	8.5–10.2	mg/dL
Albumin Level	3.4	Normal	3.4–5.4	g/dL
Total Protein	6.8	Normal	6.0–8.3	g/dL
Sodium Level	138	Normal	135–145	mEq/L
Potassium Level	4.9	Normal	3.7–5.2	mEq/L
Chloride Level	105	Normal	96–106	mEq/L
CO_2_ Level	24	Normal	23–29	mEq/L
Blood Urea Nitrogen (BUN)	51	↑ (High)	6–20	mg/dL
Glucose	193	↑ (High)	70–100 (Fasting)	mg/dL
Total Bilirubin	0.3	Normal	0.1–1.2	mg/dL
Alkaline Phosphatase	92	Normal	20–130	U/L
SGOT (AST)	37	Normal	8–33	U/L
SGPT (ALT)	53	↑ (High)	4–36	U/L
Creatinine	2.58	↑ (High)	M: 0.74–1.35; F: 0.59–1.04	mg/dL
eGFR Estimation	21	↓ (Low)	>60	mL/min/1.73 m^2^

**Table 2:** Complete blood count with differential analysis from day 6 of admission

Test Parameter	Result	Direction of Change	Adult Reference Range	Units
RBC Count	3.87	Normal	Men: 4.35–5.65; Women: 3.92–5.13	Million cells/μL
Hemoglobin	10.7	↓ (Low)	Men: 13.5–17.5; Women: 12.0–15.5	g/dL
Hematocrit	32.4	↓ (Low)	Men: 40–54%; Women: 36–48%	%
MCV (Mean Corpuscular Volume)	83.7	Normal	80–100	fL
MCH (Mean Corpuscular Hemoglobin)	27.6	Normal	27–31	pg/cell
MCHC (Mean Corpuscular Hemoglobin Concentration)	33	Normal	32–36	g/dL
RDW (Red Cell Distribution Width)	15.5	↑ (High)	12.0–15.0	%
Platelet Count	503	↑ (High)	150–450	×103/μL
MPV (Mean Platelet Volume)	9.4	Normal	7.5–12.0	fL
Neutrophils	56.6	Normal	40–60	%
Lymphocytes	27	Normal	20–40	%
Monocytes	13.2	↑ (High)	2–8	%
EOS Count (Eosinophils)	1.8	Normal	1–4	%
Basophils	0.6	Normal	0.5–1.0	%
Other Cells on Differential	0.8	N/A	N/A	%
Immatures Grans	0.8	N/A	N/A	%

## Discussion

This case highlights the diagnostic and therapeutic challenges of evaluating severe gastrointestinal intolerance in a patient with type 2 diabetes mellitus on tirzepatide therapy, augmented by daily cannabis use, and multiple comorbidities. GLP-1 receptor agonists have become increasingly central to type 2 diabetes management, with tirzepatide, an agent with dual incretin activity (gastric inhibitory polypeptide (GIP)/GLP-1 agonism), showing superior weight loss and glycemic control compared to other GLP-1 analogs [4]. However, these benefits are frequently accompanied by dose-dependent gastrointestinal adverse events, including nausea, vomiting, and diarrhea, which may be severe enough to warrant discontinuation [5].

Our patient’s presentation of recurrent, intractable vomiting shortly after dose escalation of tirzepatide is consistent with the established temporal relationship of GLP-1 dose titration and gastrointestinal (GI) side effects. Importantly, her daily cannabis use added diagnostic complexity, as cannabis hyperemesis syndrome can present with nearly identical symptoms. Differentiating between drug-induced gastroparesis, incretin-related intolerance, and cannabis hyperemesis syndrome remains a clinical challenge, particularly in patients with chronic multimorbidity and polypharmacy [7,8]. The Naranjo scale is a causality assessment method for assessing all forms of drug-induced adverse events, and accordingly to our data, a score of 5/13 indicates a probable adverse drug reaction apropos causality of nausea and vomiting after utilization of glucagon-like peptide-1 receptor agonists (GLP1-RAs) [9].

Additionally, her course was complicated by metabolic derangements, hemodynamic instability, and gastrointestinal bleeding. While tirzepatide has not been strongly associated with GI bleeding, delayed gastric emptying may predispose to mucosal irritation or exacerbate pre-existing gastrointestinal pathology. Her history of pancreatitis is also notable, as GLP-1 receptor agonists have been scrutinized for potential associations with pancreatitis, although causality remains debated [3].

This case underscores the importance of a multidisciplinary approach, including endocrinology, gastroenterology, and cardiology, in managing complex adverse drug reactions. Careful monitoring during GLP-1 titration, especially in patients with advanced CKD, cardiovascular disease, or prior GI pathology, is essential. For GLP1-RAs such as Ozempic®, it is recommended to start dosing at 0.25 mg weekly, escalating to a maintenance dose of 0.5 mg weekly at week 5, with an option to escalate the maintenance dose to 1 mg weekly after a minimum of four weeks on 0.5 mg if additional glycemic control is merited. Moreover, Ozempic® can be escalated to a maintenance dose of 2 mg weekly after a minimum of four weeks on 1 mg dosing if glycemic parameters are still not well controlled. Similarly, Wegovy is recommended to initiate dosing at 0.25 mg weekly; however, maintenance dosing is recommended to be escalated to 2.4 mg weekly at week 17 as recommended by Vannabouathong et al. (2022) [10].

In an open-label Israeli trial involving roughly 100 adults with type 2 diabetes, patients were randomized to compare standard semaglutide titration (starting at 0.25 mg/week with dose doubling every four weeks to reach 1 mg/week) versus a much slower, flexible escalation (beginning at 0.0675 mg/week and increasing by 0.0675 mg each week until 1 mg/week, with an extra week added before any increase if side effects emerged). Over six months, only 2% of those in the slow-titration arm discontinued therapy due to gastrointestinal adverse effects, versus 19% in the label-recommended group; participants on the slower schedule also experienced significantly less nausea and asthenia. Despite the markedly different ramp-up speeds, both groups achieved an average dose of ≈1 mg/week by month 6 and demonstrated equivalent improvements in body weight and glycemic control [11]. Clinicians should also screen for concurrent cannabis use, as it may mimic or worsen GLP-1-related side effects, delaying appropriate management.

## Conclusions

Tirzepatide represents a major advance in type 2 diabetes management, but this case emphasizes that its gastrointestinal adverse effects can be severe, particularly after dose escalation in high-risk patients. Cannabis use further complicates the diagnostic process, raising the possibility of overlapping etiologies for intractable vomiting. Early recognition, medication review, and a multidisciplinary care strategy are critical for distinguishing between GLP-1 intolerance, cannabis hyperemesis, and other gastrointestinal disorders. Ultimately, careful patient selection, slower titration, and vigilance for red-flag symptoms may improve safety and outcomes in the expanding use of incretin-based therapies in the setting of increased cannabis prevalence and usage.
